# Oleaginous yeasts from Ethiopia

**DOI:** 10.1186/s13568-016-0242-8

**Published:** 2016-09-15

**Authors:** Tamene Milkessa Jiru, Dawit Abate, Nicholas Kiggundu, Carolina Pohl, Marizeth Groenewald

**Affiliations:** 1 Microbial, Cellular and Molecular Biology Department, College of Natural Sciences, Addis Ababa University, P.O. Box: 1176, Addis Ababa, Ethiopia; 2 Department of Agricultural and Biosystems-Engineering, School of Food Technology, Nutrition and Bio-Engineering, Makerere University, P.O. Box: 7062, Kampala, Uganda; 3 Department of Microbial, Biochemical and Food Biotechnology, College of Agricultural Sciences, University of the Free State, P.O. Box: 339, Bloemfontein, South Africa; 4 CBS-KNAW Fungal Biodiversity Center, Uppsalalaan 8, P.O. Box: 85167, 3584 CT Utrecht, The Netherlands

**Keywords:** Biodiesel, Oleaginous yeast, Single cell oil, Yeast identification

## Abstract

Oleaginous microorganisms can produce high amounts of oil (>20 % of their biomass) under suitable cultivation conditions. In this research work 200 samples were collected from soil, plant surfaces (leaves, flowers and fruits), waste oils from traditional oil milling houses and dairy products (cheese, milk and yoghurt) in Ethiopia. Three hundred and forty yeast colonies were isolated from these samples. By applying Sudan III staining tests, 18 strains were selected as possible oleaginous yeasts. The 18 strains were identified and characterized for their lipid production as a feedstock for biodiesel production in the future. They were identified using morphological and physiological methods as well as sequencing the 3′end of the small-subunit rRNA gene, the internal transcribed spacer regions (ITS; ITS 1, ITS 2 and the intervening 5.8S rRNA gene), and the D1/D2 domain of the 26S rRNA gene. The 18 yeasts were identified as *Cutaneotrichosporon curvatus* (syn, *Cryptococcus curvatus*) (PY39), *Rhodotorula kratochvilovae* (syn, *Rhodosporidium kratochvilovae*) (SY89), *Rhodotorula dairenensis* (SY94) and *Rhodotourula mucilaginosa* (SY09, SY18, SY20, PY21, PY23, PY25, SY30, PY32, SY43, PY44, SY52, PY55, PY61, SY75 and PY86). Under nitrogen-limited cultivation conditions, *R. mucilaginosa* PY44 produced the highest biomass (15.10 ± 0.54 g/L), while *R. mucilaginosa* PY32 produced the lowest biomass (10.32 ± 0.18 g/L). The highest lipid yield of 6.87 ± 0.62 g/L and lipid content of 46.51 ± 0.70 % were attained by *C. curvatus* (syn, *C. curvatus*) PY39. On the other hand, *R. mucilaginosa* PY61 gave the lowest lipid yield (2.06 ± 0.52 g/L) and *R. mucilaginosa* SY52 gave the lowest lipid content of 16.99 ± 0.85 %. The results in this research work suggest that much more oleaginous yeasts can be isolated from Ethiopian environment. On the basis of their substantial lipid production abilities, the three oleaginous yeast strains PY39, SY89 and SY18 were selected and recommended for further optimization processes.

## Introduction

Oleaginous microorganisms can produce high amounts of oil (>20 % of their biomass) under suitable cultivation conditions (Wynn and Ratledge 2006). These types of organisms can belong to yeasts, filamentous fungi, microalgae and bacteria. They are of great interest due to their ability to accumulate high amounts of lipids in separate lipid bodies (Drucken 2008; Li et al. 2008), high lipid production rate, their relatively high growth rates and the resemblance of their TAGs to plant oils (Pan et al. 2009; Kosa and Ragauskas 2010).Their production is not influenced by external factors such as their origin, season or climatic changes (ThiruM and Rangaswamy 2011). However, for the maximum production of lipid by oleaginous microorganisms, the culture medium has to be provided with an excess of carbon source and limited amount of nitrogen sources (Ratledge 2004). The best studied oleaginous yeasts belong to the in genera *Candida*, *Cryptococcus*, *Lipomyces*, *Rhodosporidium*, *Rhodotorula, Trichosporon and Yarrowia* (Pan et al. 2009). All except *Lipomyces* and *Yarrowia* are basidiomycetous yeasts. Many basidiomycetous yeast species including oleaginous ones have been included now in other existing or new genera (Liu et al. 2015; Wang et al. 2015). Accordingly, *Cryptococcus* genera have been transferred to *Cutaneotrichosporon*. Accordingly, the oleaginous yeast strain *Cryptococcus curvatus* is renamed as *Cutaneotrichosporon curvatus* (Liu et al. 2015). Similarly, *Rhodosporidium* has been transferred to *Rhodotorula* and the oleaginous yeast *Rhodosporidium kratochvilovae* is renamed as *Rhodotorula kratochvilovae* (Wang et al. 2015).

The accumulated oil (stored TAGs) in the cells of oleaginous microorganisms act as a carbon store to maintain essential metabolic processes in the event of subsequent carbon starvation (Anderson and Wynn 2001). It is also suggested that in case of marine microorganisms the accumulation of large lipid droplets in the cytosol acts as an aid to buoyancy (Anderson and Wynn 2001). The oil produced can be investigated and exploited as alternative sources of oils and fats for human consumption (Ratledge 2005) and can also be used as feedstocks for biodiesel production (Li et al. 2008).The objective of this study was to characterize and identify oleaginous yeasts from natural sources in Ethiopia and produce single cell oil as a feedstock for biodiesel production in the future.

## Materials and methods

### Sample collection and yeast isolation

Two hundred samples were collected from soil, dairy products (cheese, yogurt and whey), plant surfaces (flower, leaves, roots, fruits) and wastes from traditional oil mill houses. This was done to increase the chance of obtaining oleaginous yeasts. All samples were placed into properly labeled sterile bottles and transported to the laboratory and kept at 4 °C for further use.

A mass of 1 g of each sample was aseptically milled, ground or homogenized and added into 50 mL sterile yeast malt extract (YM) broth (10 g/L glucose, 3 g/L yeast extract, 3 g/L malt extract and 5 g/L peptone) in a 250 mL Erlenmeyer flask, and incubated at 30 °C for 24 h with shaking speed at 200 rpm so that the yeasts that are present would be enriched to a greater number (Yarrow 1998).

From these mixtures, 1 mL was added to 9 mL of distilled water and tenfold dilutions were made, ranging from 10^−1^ to 10^−5^. 0.1 mL from each dilution was spread onto YM agar plates. Chloramphenicol (1 g/L) and sodium propionate (2.5 g/L) were added to the media to inhibit bacterial growth and reduce the growth of filamentous fungi, respectively.

### Characterization and identification of oleaginous yeasts

#### Screening for oleaginous yeasts

The isolated yeast cultures were further screened for their lipid producing abilities by qualitative analysis with the Sudan III staining technique (Thakur et al. 1988; Xing et al. 2012). Those yeasts which tested positive for lipid production capacity were characterized and identified.

#### Morphological and physiological characterization of oleaginous yeasts

Traditional identification of the oleaginous yeast strains was based on the comparative analysis of distinguishable morphological characteristics of the cultures, the presence or absence of sexual reproduction and different physiological characteristics of the isolates studied. The results were compared with the criteria set by Yarrow (1998); Barnett et al. (2000); Kurtzman et al. (2003, 2011). Morphological characteristics of the cultures such as colony color, size, shape, elevation, texture and diameter were studied and characterized. Cell shape, dimension and budding features of the strains grown in a liquid YM medium were observed using light microscopy. The formation of pseudohyphae, ballistoconidia and ascospore were also tested following the procedures of Yarrow (1998); Barnett et al. (2000); Kurtzman et al. (2003, 2011).

The physiological tests that were done include fermentation of carbon sources, assimilation of carbon and nitrogen sources using auxonographic method, urea hydrolysis, diazonium blue B color reaction, growth in media of high osmotic pressure (60 % glucose), degradation of fat (lipolytic activity) and formation of extracellular amyloid compounds or starch formation.

#### Molecular characterization of oleaginous yeasts

Genomic DNA was extracted from cultures grown on 40 g/L glucose, 5 g/L peptone, 5 g/L yeast autolysate, and 20 g/L agar (GPYA) medium for three days using the FastDNA kit (BIO101, Carlsbad, CA, U.S.A.) with the “FastPrep” Instrument (Q-Biogene). Primers V9G (de Hoog and Gerrits van den Ende 1998) and LR5 (Vilgalys and Hester 1990) were used to amplify the region of the rRNA gene operon that includes the 3′ end of the small-subunit rRNA gene, the ITS regions (ITS 1, ITS 2 and the intervening 5.8S rRNA gene), and the D1/D2 domains of the 26S rRNA gene of the large subunit, as described by Knutsen et al. (2007).

The PCR products were separated by electrophoresis at 80 V for 40 min on a 0.8 % (w/v) agarose gel containing 0.1 μg/mL ethidium bromide in 1× TAE buffer (0.4 M Tris, 0.05 M NaAc and 0.01 M EDTA, pH 7.85) and examined under UV-light. The amplicons were sequenced in both directions using the primers LR0R (Vilgalys and Hester 1990) and LR5 for the D1/D2 domain, while the primers V9G and ITS4 (White et al. 1990) were used for the ITS domain (ITS 1, ITS 2 and the intervening 5.8S rRNA gene). The BigDye Terminator version 3.1 Cycle Sequencing kit (Applied Biosystems) was used according to the manufacturer’s recommendations and the products were analyzed on an ABI Prism 3730XL DNA Sequencer (Perkin-Elmer). A consensus sequence was computed from the forward and reverse sequences with SeqMan version 8 from the Lasergene package (DNASTAR). All sequences of the studied strains were blasted against sequences in GenBank (http://blast.ncbi.nlm.nih.gov/Blast.cgi) and the CBS yeast database (http://www.cbs.knaw.nl/Collections/) in order to identify the oleaginous yeasts. Sequences of theD1/D2 of the 26S rRNA obtained during this study and related sequences from the GenBank (NCBI) database were aligned and phylogenetic analyses were done using MEGA 7 version (Kumar et al. 2016). The phylogenetic relationship of these yeast strains is displayed in a distance based Neighbor-Joining tree.

#### Cultivation of oleaginous yeast in nitrogen-limited medium

Those yeast isolates tested positive for Sudan III stain were cultivated on nitrogen-limited media containing (g/L):glucose (70), (NH_4_)_2_SO_4_ (0.30), yeast extract (0.30), KH_2_PO_4_ (2.0), MgSO_4_·7H_2_O (1.5), citric acid (0.25), CaCl_2_2H_2_O (0.1), FeSO_4_·7H_2_O (0.035), ZnSO_4_·7H_2_O (0.011), MnSO_4_·H_2_O (0.007), CoCl·6H_2_O (0.002), Na_2_MoO_4_·2H_2_O (0.0013) and CuSO_4_·5H_2_O (0.001). An inoculum size of 5 % (v/v) (~7.94 × 10^8^cells/mL). Cultures were performed in 250 mL conical flasks containing 50 mL medium. The oleaginous yeast strains were cultured in this nitrogen-limited media for 144 h at 30 °C on a rotary shaker at 200 rpm. The initial pH of the medium was 5.5.

### Determination yeast biomass

For lipid extractions and to determine the total weight of the lipid content of the cells, yeast cells were harvested by centrifugation at 5000×*g* for 15 min. The supernatants were discarded. The pellet was harvested and washed twice with distilled water and frozen at −80 °C. The cells were then freeze dried over night to constant weight and the dry weight determined gravimetrically (Pan et al. 2009).

### Lipid content determination

Lipid extraction was done following Folch et al. (1957) with some modifications. Freeze dried biomass was ground with a pestle in a mortar. Then 1 g of sample was homogenized with 3.75 mL solvent mixture of chloroform and methanol (2:1) and was left overnight at room temperature. The following day the solvent mixture was transferred into clean separating funnel through Whatman No 1 filter paper. Then 1.25 mL of solvent mixture was poured through filter paper into separating funnel. This was followed by washing with 0.75 mL of distilled water. The solvent/water mixture was left overnight to separate into two clear phases. The bottom phase was collected and the solvents evaporated under vacuum. Diethyl ether was used to transfer the extract into pre-weighed glass vials. The glass vials were left overnight in a fume hood to evaporate the diethyl ether. The following day, the vial with the sample was weighed. From this lipid yield and lipid content were calculated.

$${\text{Single cell oil content}} = {{{\text{Single cell oil weight }} ( {{\text{g}}/{\text{L}}})}/ {{\text{Cell dry weight }} ( {{\text{g}}/{\text{L}}}) \times 100}}$$

### Statistical analysis

One way-ANOVA was performed to calculate significant differences in treatment means. SPSS version 20.0 software was used for interpretation of data. Data with a *p* value <0.05 were considered significantly different. All experiments were done in triplicate.

Each oleaginous yeast strain has given a GenBank accession number (ACC. NO.) as follows. SY94 (ACC. NO. KX525689); PY23 (ACC. NO. KX525690); PY86 (ACC. NO. KX525691); SY20 (ACC. NO. KX525692); SY43 (ACC. NO. KX525693); PY21 (ACC. NO. KX525694); SY09 (ACC. NO. KX525695); SY75 (ACC. NO. KX525696); PY25 (ACC. NO. KX525697); PY44 (ACC. NO. KX525698); PY5 (ACC. NO. KX525699); SY30 (ACC. NO. KX525700); PY61 (ACC. NO. KX525701); SY18 (ACC. NO. KX525702); SY89 (ACC. NO. KX525703); PY39 (ACC. NO. KX525704) and PY32 (ACC. NO. KX525705).

## Results

### Screening for oleaginous yeasts

**Fig. 1:**
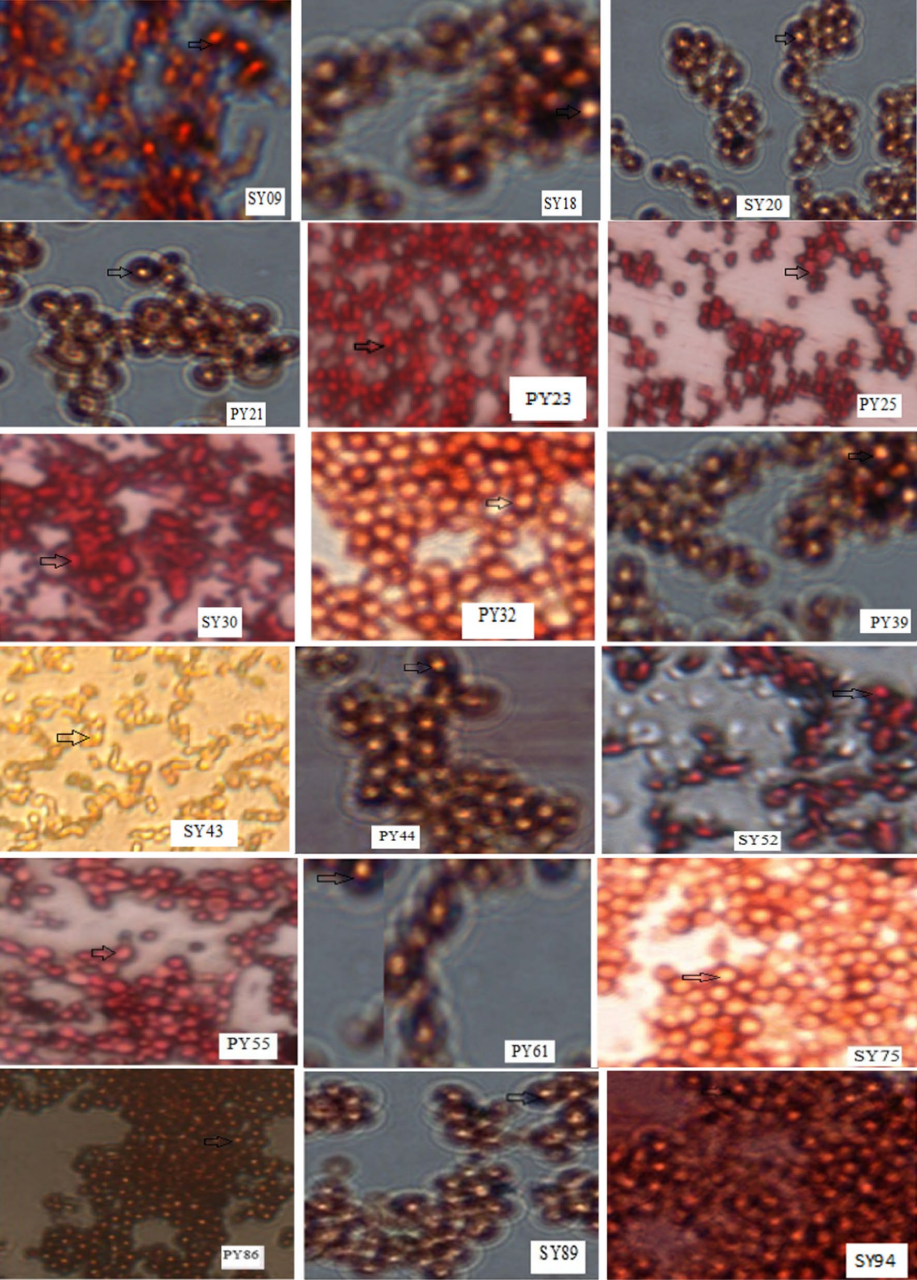
Photomicrographs of Sudan III stained cells of the selected oleaginous yeasts. Lipid bodies stained *yellow*

From the different samples obtained, 340 isolates with the morphology typical of yeast were obtained. Each of these isolates was screened for their lipid producing potential. On the basis of screening results from Sudan III staining technique, 18 strains were selected. When cells of these yeasts stained with Sudan III stain and observed under oil immersion objectives, yellow intracellular inclusions were seen in each case (Fig. 1) and based on the above observations 18 oleaginous yeast strains were selected for further analyses.

### Physiological and morphological analyses

Despite advances in molecular identification techniques, morphological and physiological characteristics are still of great importance in yeast systematics and identification. In this study, the already screened yeast strains were characterized and identified with different traditional methods (morphological and physiological) before proceeding to molecular identification. The results regarding the colony morphology, budding patterns and cell shapes are indicated in Table 1.

**Table 1:** Colony characteristics, budding features and shape of selected yeasts

Strain	Source	Colony morphology(colony color, size, shape, texture and diameter)	Budding pattern	Cell shape
SY09	Soil	Orange, medium, circular, smooth, dry colonies with diameter 3–5 mm	Bipolar	Ovoidal to spherical
SY18	Soil	Pink, small, circular, smooth, dry colonies with diameter 2–3 mm	Multipolar	Ovoidal to spherical
SY20	Soil	Orange, small, circular, smooth, moist colonies with diameter 2–3 mm	Multipolar	Ovoidal to spherical shape
SY30	Soil	Pink, medium, circular, smooth, moist colonies with diameter 3–5 mm	Multipolar	Ovoidal to spherical
SY43	Soil	Orange, irregular, large, smooth, moist colonies with diameter of 5–10 mm	Multipolar	Ovoidal to spherical
SY52	Soil	Salmon, medium, irregular, smooth, dry colonies with diameter of 3–5 mm	Bipolar	Ovoidal to spherical
SY75	Soil	Orange, medium, irregular, wrinkled, moist colonies with diameter 3–5 mm	Unipolar	Ovoidal to spherical
SY89	Soil	Salmon, medium, circular, smooth, dry colonies with diameter of 3–5 mm	Bipolar	Ovoidal
SY94	Soil	Orange, large, irregular, wrinkled, dry colonies with diameter of 5–10 mm	Multipolar	Ovoidal
PY21	Fruit	Orange, small, circular, smooth, moist colonies with diameter of 2–3 mm	Multipolar	Ovoidal to spherical
PY23	Fruit	Pink, circular, medium, smooth, moist colonies with diameter of 3–5 mm	Multipolar	Ovoidal to spherical
PY25	Fruit	Orange, circular, medium, smooth, dry colonies with 3–5 mm in diameter	Multipolar	Ovoidal to spherical
PY32	Flower	Orange, circular, medium, smooth, dry colonies with diameter of 3–6 mm	Multipolar	Ovoidal to spherical
PY39	Flower	Creamy, large, irregular, wrinkled, moist, colonies with diameter of 5–10 mm	Multipolar	Ovoidal to spherical
PY44	Flower	Pink, circular, medium, smooth, dry colonies with diameter of 3–5 mm	Multipolar	Ovoidal to spherical
PY55	Flower	Pink, medium, circular, wrinkled, moist colonies with diameter of 3–5 mm	Multipolar	Ovoidal
PY61	Leaf	Red, medium, irregular, wrinkled, moist colonies with diameter of 3–5	Unipolar	Ovoidal to spherical
PY86	Leaf	Salmon, small, irregular, wrinkled, moist colonies with diameter of 2–3 mm	Bipolar	Ovoidal

Other morphological tests that were carried out for identification purposes were test for the formation of pseudohyphae, ballistoconidia and ascospore. The test results are shown in Table 2. The results obtained from the physiological tests done are also listed in Table 2.

**Table 2:** Other phenotypic characters of selected yeasts

Yeast strain	Ballistoconidia	Ascospore formation	Pseudohyphae	DBB color reaction	Fat splitting	Urea hydrolysis	Growth in 60 % glucose	Starch formation	Glucose fermentation	Assimilation test											
										d-Glucose	Sucrose	Maltose	Lactose	d-Galactose	Trehalose	Melibiose	Celobiose	Peptone	KNO_3_	l-Lysine	Creatine
SY09	−	−	+	+	−	+	−		−	+	+	+	−	+	+	−	+	+	−	+	−
SY18	−	−	+	+	−	+	−	−	−	+	+	+	−	+	+	−	−	+	−	+	−
SY20	−	−	+	+	−	+	−	−	−	+	+	+	−	+	+	−	−	+	−	+	−
SY30	−	−	−	+	−	+	−	−	−	+	+	+	−	+	+	−	−	+	−	+	−
SY43	−	−	+	+	−	+	−	−	−	+	+	+	−	+	+	−	−	+	−	+	−
SY52	−	−	+	+	−	+	−	−	−	+	+	+	−	+	+	−	−	+	−	+	−
SY75	−	−	+	+	−	+	−	−	−	+	+	+	−	+	+	−	−	+	−	+	−
SY89	−	−	+	+	−	+	−	−	−	+	+	+	−	+	+	+	+	+	+	−	−
SY94	−	−	+	+	−	+	−	−	−	+	+	+	−	+	+	+	+	+	+	+	−
PY21	−	−	−	+	−	+	−	−	−	+	+	+	−	+	+	−	−	+	−	+	−
PY23	−	−	−	+	−	+	−	−	−	+	+	+	−	+	+	−	−	+	−	+	−
PY25	−	−	+	+	−	+	−	−	−	+	+	+	−	+	+	−	−	+	−	+	−
PY32	−	−	+	+	−	+	−	−	−	+	+	+	−	+	+	−	+	+	−	+	−
PY39	−	−	+	+	+	+	−	+	−	+	+	+	+	+	+	+	−	+	−	+	−
PY44	−	−	−	+	−	+	−	−	−	+	+	+	−	+	+	−	−	+	−	+	−
PY55	−	−	+	+	−	+	−	−	−	+	+	+	−	+	+	−	−	+	−	+	−
PY61	−	−	−	+	−	+	−	−	−	+	+	+	−	+	+	−	−	+	−	+	−
PY86	−	−	+	+	−	+	−	−	−	+	+	+	−	+	+	−	+	+	−	+	−

Morphological and physiological characterization test results lead to the identification of isolated and screened oleaginous yeasts at the genus level. Based on this SY09, SY18, SY20, SY30, SY43, SY52, SY75, PY21, PY23, PY25, PY32, PY44, PY61, PY86 and SY94 were assigned as *Rhodotorula* species. SY89 was assigned as *Rhdodotorula* (syn, *Rhodosporidium*) species. Furthermore, PY39 was classified as *Cutaneotrichosporon* (syn, *Cryptococcus*) or *Trichosporon* species.

### Molecular characterization of oleaginous yeasts

**Fig. 2:**
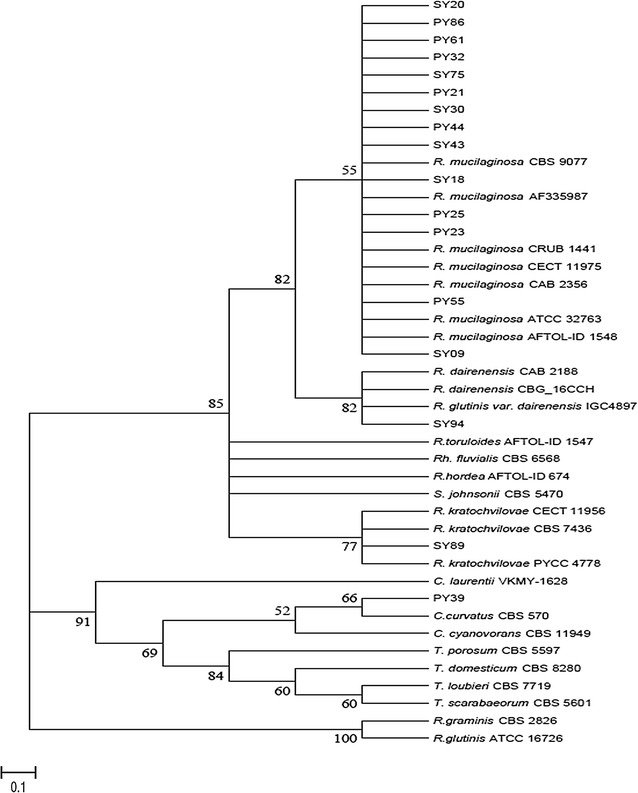
Phylogenetic tree of the D1/D2 domain of 26S rRNA gene sequences of oleaginous yeast strains with related yeast species in NCBI database. The tree was constructed using the Neighbor-Joining method of MEGA 7 software. The optimal tree with the sum of branch length = 1.81465379 is shown. Bootstrap values (1000 tree interactions) are indicated at the nodes. Each of the strains have the following accession numbers. SY94 (ACC. NO. KX525689); PY23 (ACC. NO. KX525690); PY86 (ACC. NO. KX525691); SY20 (ACC. NO. KX525692); SY43 (ACC. NO. KX525693); PY21 (ACC. NO. KX525694); SY09 (ACC. NO. KX525695); SY75 (ACC. NO. KX525696); PY25 (ACC. NO. KX525697); PY44 (ACC. NO. KX525698); PY55 (ACC. NO. KX525699); SY30 (ACC. NO. KX525700); PY61 (ACC. NO. KX525701); SY18 (ACC. NO. KX525702); SY89 (ACC. NO. KX525703); PY39 (ACC.NO. KX525704) and PY32 (ACC. NO. KX525705)

After screening yeast strains with Sudan III, morphological and physiological characterization and identification of such potential oleaginous yeasts, they were further identified by sequence analysis of the ITS and the D1/D2 domains of the 26S rRNA gene and the sequences obtained from this study and that of closely related sequences from GenBank are displayed in Fig. 2.

The sequences were blasted against sequences in GenBank (NCBI) (http://blast.ncbi.nlm.nih.gov/Blast.cgi) and the CBS database (http://www.cbs.knaw.nl/Collections/). The percentage of sequence similarity with data of additional strains and especially type strains were determined. The D1/D2 sequences were aligned and the phylogenetic relationship of these yeast strains is displayed in a distance based Neighbor-Joining tree (Fig. 2). The tree obtained by analyzing ITS domain is not shown here since the same relationship among species were obtained. SY52 failed to be sequenced using D1/D2 domains of the 26S rRNA gene so that it is omitted in the phylogeny. However, it was identified as *R. mucilaginosa* species based on morphological and physiological tests as well as by comparing their ITS sequences against sequences in GenBank (http://blast.ncbi.nlm.nih.gov/Blast.cgi) and the CBS yeast database (http://www.cbs.knaw.nl/Collections/) (data not shown). On the other hand, the D1/D2 domain of 26S rRNA gene of PY39 (ACC. NO. KX525704) and *Cutaneotrichosporon curvatus* (*C. curvatus)* CBS 570 shared 99 % similarity. The sequence analysis results of SY89(ACC. NO. KX525703) also revealed that the amplified sequences were 100 % identical to those corresponding to *Rhodotorula kratochvilovae* (*R. kratochvilovae*) CBS 7436 and *R. kratochvilovae* CECT 11956, while 99 % similarity was exhibited by this strain with *R. kratochvilovae* PYCC 4778. In addition, the sequence analysis result showed that the amplified sequences of SY94 were 99 % identical to those corresponding to *Rhodotorula dairenensis* (*R. dairenensis*) CAB 2188, *R. dairenensis* CBG_16CCH, *R. glutinis var. dairenensis* IGC4897. Furthermore, strains PY21 (ACC. NO. KX525694), PY23 (ACC. NO. KX525690), PY25 (ACC. NO. KX525697), PY32 (ACC. NO. KX525705) PY44 (ACC. NO. KX525698), PY55 (ACC. NO. KX525699), PY61 (ACC. NO. KX525701), PY86 (ACC. NO. KX525691), SY09 (ACC. NO. KX525695), SY18 (ACC. NO. KX525702), SY20 (ACC. NO. KX525692), SY30 (ACC. NO. KX525700), SY43 (ACC. NO. KX525693) and SY75 (ACC. NO.KX525696) were 100 % identical to *Rhodotorula mucilaginosa* (*R.mucilaginosa*) CAB 2356, *R. mucilaginosa* ATCC 32763, *R.mucilaginosa* AFTOL-ID 1548, *R.mucilaginosa* CECT11975, *R.mucilaginosa* CRUB 1441, *R.mucilaginosa* AF335987 and *R. mucilaginosa* CBS 9077.

### Analyses of lipid production

In order to select the best oleaginous yeast from the screened 18 oleaginous yeasts, the lipid production yield and cellular lipid content were evaluated after 144 h of incubation of each oleaginous yeast in a nitrogen-limited culture media at 30 °C (Table 3). The yeast strain PY44 gave the most biomass (15.10 ± 0.54 g/L), whereas PY32 produced the least biomass (10.32 ± 0.18 g/L).Maximum lipid yield was attained by PY39 (6.87 ± 0.62 g/L) followed by SY89 (5.79 ± 0.30 g/L), SY18 (5.27 ± 0.27 g/L) and SY09 (4.97 ± 0.27 g/L). PY61 gave the lowest lipid yield (2.06 ± 0.52 g/L). On the other hand, maximum cellular lipid content was attained by PY39 (46.51 ± 0.70 %) followed by SY89 (39.33 ± 0.57 %), SY18 (38.61 ± 0.39 %) and SY09 (36.15 ± 0.41 %). SY52 had the lowest lipid content, i.e., 16.99 ± 0.85 %. As it can be seen from Table 3, both SY94 and PY21 gave good lipid yield and cellular lipid content results. On the basis of their substantial lipid production abilities, the three oleaginous yeast strains PY39, SY89 and SY18 were selected and recommended for further optimization processes.

**Table 3:** Biomass production, lipid yield and lipid content of 18 oleaginous yeast strains under nitrogen-limited medium

Strain	Dry biomass (g/L)	Lipid yield (g/L)	Lipid content (%)
*R. mucilaginosa* SY09	13.75 ± 0.77^abcd^	4.97 ± 0.27^bcd^	36.15 ± 0.41^c^
*R. mucilaginosa* SY18	13.65 ± 0.86^abcd^	5.27 ± 0.27^abc^	38.61 ± 0.39^b^
*R. mucilaginosa* SY20	10.79 ± 1.24^ef^	2.96 ± 0.81^def^	27.43 ± 0.60^e^
*R. mucilaginosa* SY30	11.74 ± 0.64^def^	3.10 ± 0.45^def^	26.40 ± 0.45^f^
*R. mucilaginosa* SY43	12.56 ± 0.45^cde^	3.39 ± 0.29^cde^	26.99 ± 0.35^e^
*R. mucilaginosa* SY52	13.65 ± 0.70^abcd^	2.32 ± 0.47^fg^	16.99 ± 0.85^k^
*R. mucilaginosa* SY75	12.60 ± 0.66^cde^	3.03 ± 0.16^def^	24.05 ± 0.42^fg^
*R.kratochvilovae* SY89	14.72 ± 0.38^abc^	5.79 ± 0.30^ab^	39.33 ± 0.57^b^
*R. dairenensis* SY94	12.95 ± 0.72^bcd^	4.33 ± 0.70^bcde^	33.44 ± 0.74^d^
*R. mucilaginosa* PY21	12.70 ± 1.02^bcde^	4.35 ± 0.61^bcde^	34.26 ± 0.55^cd^
*R. mucilaginosa* PY23	13.58 ± 0.79^abcd^	3.06 ± 0.52d^ef^	22.54 ± 0.66^h^
*R. mucilaginosa* PY25	10.32 ± 0.33^f^	2.19 ± 0.69^fg^	21.22 ± 0.51^i^
*R. mucilaginosa* PY32	10.02 ± 0.18^g^	2.34 ± 0.42^fg^	23.35 ± 0.37^g^
*C. curvatus* PY39	14.77 ± 0.65^ab^	6.87 ± 0.62^a^	46.51 ± 0.70^a^
*R. mucilaginosa* PY44	15.10 ± 0.54^a^	4.11 ± 0.60^cde^	27.23 ± 0.55^e^
*R. mucilaginosa* PY55	12.66 ± 1.21^bcde^	2.49 ± 0.48^fg^	19.67 ± 0.28^j^
*R. mucilaginosa* PY61	10.22 ± 0.43^f^	2.06 ± 0.52^g^	20.16 ± 0.71^j^
*R. mucilaginosa* PY86	13.76 ± 0.69^abcd^	3.36 ± 0.56^cde^	24.42 ± 0.71^fg^

All parameters are expressed as mean ± SD values in the same column,* n* = 3, within each column, means are significantly different (*p* < 0.05), unless they have a common letter

## Discussion

Yeasts are a polyphyletic group of basidiomycetous and ascomycetous fungi with the ability to grow unicellularly. This diverse group of microorganisms represents a part of the microbiota in all natural ecosystems, such as soils, freshwaters and marine waters from the ocean surface to the deep sea (Kutty and Philip 2008). They are widely distributed in the different natural ecosystems and environments. Some yeasts also colonize more extreme environments, such as low temperatures, low oxygen availabilities, and oceanic waters (Butinar et al. 2007). Currently there are more than 2000 recognized yeast species (http://www.mycobank.org). Yeasts grow typically in moist environments where there is an abundant supply of sugar-rich sources. They are common inhabitants of leaf, fruit surfaces and roots as well as various types of food. Few types of yeast have the capacity to degrade polymers like starch and cellulose. Yeasts are found in the atmosphere as well as certain restricted habitats. Some yeasts inhabit both agricultural and forest soil (Sláviková and Vadkertiová 2003). They also occur in the intestinal flora of mammals and some insects (Martini 1992).

In a similar fashion, oleaginous yeasts have been isolated from diverse natural habitats such as various soils, water bodies and flower surfaces (Breuer and Harms 2006; Dai et al. 2007; Pan et al. 2009; Amaretti et al. 2010).

In the current study, most of the screened and identified oleaginous yeasts were isolated from soil (9), flower (4), fruit (3) and leaf (2) (Table 1). So that soil can be a good source for isolation of oleaginous yeasts.

Conventionally the presence of lipids from microorganisms is estimated using Sudan Black B or Sudan III (Thakur et al. 1988) or Nile red (Kimura et al. 2004) staining. These techniques only provide preliminary information and do not allow precise insight into intracellular lipid content of the lipid accumulation ability of the tested microorganism. So that there may be a high proportion of false-positive is results with respect to lipid content (Pan et al. 2009).

After screening the potential oleaginous yeasts, identification and characterization was undertaken using traditional (morphological and physiological) as well as molecular methods. Morphological and physiological characterization test results were then compared with the criteria set by Yarrow (1998), Barnett et al. (2000), Kurtzman et al. (2003, 2011) and lead to the identification of isolated and screened oleaginous yeasts at the genus level. Their identification was confirmed by sequencing both ITS domain and D1/D2 domains of the 26S rRNA gene of the large subunits of each strain.

In the current study, the 18 yeast strains were identified by sequence analysis of both ITS and D1/D2 domain of the large subunit. The tree obtained by analyzing ITS domain is not shown here since the same relationship among species was obtained. However, it has helped in identifying SY52. On the other hand, sequence analysis of D1/D2 the 26S rRNA gene of the large subunit of PY39, SY89 and SY94 indicates that these strains are species of *C. curvatus, R. kratochvilovae* and *R. dairenensis,* respectively. On the other hand the remaining oleaginous yeasts strains were identified as *R. mucilaginosa* (Fig. 2).

Sequence analysis of ITS domain and/or D1/D2 domain of the 26S rRNA gene techniques were applied in identifying yeasts for various purposes. For example, Pan et al. (2009) identified 20 oleaginous yeasts by sequencing the D1/D2 domains of 26S r DNA (rRNA gene). The nucleotide sequences of ITS 1 and 2 regions in the rRNA gene were determined by directly sequencing PCR-amplified fragments for species (17 species and five varieties) in the genus *Trichosporon* (Sugita et al. 1999). Comparative sequence analysis suggests that six medically relevant species, *T. asahii*, *T. asteroides*, *T. cutaneum*, *T. inkin*, *T. mucoides* and *T. ovoides*. Some other investigators were able differentiate the different species within *Saccharomyces* genera (Oda et al. 1997; Fernandez-Espinar et al. 2000). Fernandez-Espinar et al. (2000) identified flour yeasts using such method. Yeasts involved in the spoilage of yoghurt were also identified using this method (Caggia et al. 2001). Leaw et al. (2006) also identified medically important yeasts using ITS1 and ITS2 sequencing. They were able to identify 373 strains (86 species). Mohamed et al. (2014) identified three isolates of *R. mucilaginosa* and three of *R. glutinis* using ITS.

Biomass production and lipid accumulation of oleaginous microorganisms are affected by the carbon source available during fermentation (Mamatha 2009). For the production of maximum cell biomass and lipid yield in oleaginous yeasts, medium with an excess of carbon source and limited amount of nitrogen sources has a significant influence (Ratledge 2004). In this study, the 18 screened and identified oleaginous yeasts were further tested for their lipid production capacity by growing them in a nitrogen-limited media (Table 3). It was observed that yeasts varied in their lipid accumulating capacities with the maximum exhibited by *C. curvatus* PY39 (46.51 ± 0.70 %) and minimum exhibited by *R. mucilaginosa* PY61 (16.99 ± 0.85 % L). Other strains gave intermediate values. According to earlier works, oleaginous microorganisms differ in their ability in accumulating oil. This is the difference in their biochemistry and genetic constitution of their cells (Wynn and Ratledge 2006; Nigam and Singh 2014). The difference can be exhibited not only among different oleaginous species but among strains of the same species.

On other research work, a stain of *R. mucilaginosa* namely *R. mucilaginosa* TJY15a which was isolated from surface of marine fish was grown on hydrolysate of cassava starch. The cells of this oleaginous yeast contained 47.9 % w/w oil during batch cultivation, whereas 52.9 % (w/w) of lipid was obtained during the fed-batch cultivation (Li et al. 2010). Enshaeieh et al. (2015) tested lipid producing capacity of another strain of *R. mucilaginosa* on xylose, grass and leaves hydrolysate as substrates. Under similar conditions, *R. mucilaginosa* TJY15a accumulated 55.4 % (w/w) oil from the extract of Jerusalem artichoke tubers in its cells and cell dry weight reached 12.8 g/L within 48 h (Zhao et al. 2011). On the other hand, a strain of *R. kratochvilovae* HIMPA1 was isolated from Himalayan permafrost soil showed that boosted TAG accumulation in the lipid droplets (Patel et al. 2014). Patel et al. (2014) observed enhanced total lipid content and lipid yield of 55.56 %, 8.39 ± 0.57 g/L when the yeast was grown in Hemp seeds aqueous extract. This result was better than 41.92 %, 6.2 ± 0.8 g/L which was obtained when the yeast was grown in industrially used glucose synthetic medium. A biomass of 11 g/L and lipid content of 46 % (w/w) were exhibited by *C. curvatus* when grown on diluted (25 %) prickly-pear juice for 35 h in batch culture (Hassan et al. 1994). Another strain of this species exhibited a biomass of 69 g/L and lipid content of 48 % (w/w) of intercellular lipid when grown on a crude glycerol in a fed-batch(ThiruM and Rangaswamy 2011).

The results in this research work suggest that much more oleaginous yeasts can be isolated from Ethiopian environment. The 18 yeast strains that we isolated have the potential to be used for the production of single cell oil and hence biodiesel at the industrial level. Traditional identification methods (morphological and physiological) must be complemented with molecular method of identification especially the use of ITS and D1/D2 domains of the 26S rRNA gene sequencing. To improve the production of lipid from oleaginous yeasts, cultivation conditions for lipid production by oleaginous yeasts should be optimized, oleaginous yeasts should be subjected to mutagenesis or genetically engineered to select the best strains with the highest lipid production capacity, and cheap substrates like lignocellulosic wastes and sugar factory by-products have to be used for cultivation purpose to minimize cost of production.

## Abbreviations

ACC. NO.: accession number
TAGs: triacylglycerols

## References

[CR1] Amaretti A, Raimondi S, Sala M, Roncaglia L, Lucia MD, Leonardi A, Rossi M (2010). Single cell oils of the cold adapted oleaginous yeast *Rhodotorula glacialis* DBVPG4875. Microb Cell Fact.

[CR2] Anderson AJ, Wynn JP, Ratledge C, Kristiansen B (2001). Microbial polyhydroxyalkanoates, polysaccharides and lipids. Basic biotechnology.

[CR3] Barnett JA, Payne RW, Yarrow D (2000). Yeast-characteristics and identification.

[CR4] Breuer U, Harms H (2006). *Debaryomyceshansenii:* an extremophilic yeast with biotechnological potential. Yeast.

[CR5] Butinar L, Spencer-Martins I, Gunde-Cimerman N (2007). Yeasts in high Arctic glaciers: the discovery of a new habitat for eukaryotic microorganisms. Antonie Van Leeuwenhoek.

[CR6] Caggia C, Restuccia C, Pulvirenti A, Giudici P (2001). Identification of *Pichia anomala* isolated from yoghurt by RFLP of the ITS region. Int J Food Microbiol.

[CR7] Dai C, Tao J, Xie F, Dai Y, Zhao M (2010). Biodiesel generation from oleaginous yeast *Rhodotorula glutinis* with xylose assimilating capacity. Afri J Biotechnol..

[CR8] de Hoog GS, Gerrits van den Ende AHG (1998). Molecular diagnostics of clinical strains of filamentous basidiomycetes. Mycoses.

[CR9] Drucken Z (2008). Triacylglycerol synthesis in the oleaginous yeast *Yarrowia lipolytica*. Bioresour Technol.

[CR10] Enshaeieh M, Abdoli A, Madani M, Bayat M (2015). Recycling of lignocellulosic waste materials to produce high-value products: single cell oil and xylitol. Int J Environ Sci Technol.

[CR12] Fernandez-Espinar MT, Esteve-Zarzoso B, Querol A, Barrio E (2000). RFLP analysis of the ribosomal internal transcribed spacers and the 5.8S rRNA gene region of the genus *Saccharomyces*: a fast method for species identification and differentiation of flour yeasts. Anton van Leeuwenhoek.

[CR13] Folch J, Lees M, Sloane-Stanley GH (1957). A simple method for the isolation and purification of total lipids from animal tissues. J Biol Chem.

[CR14] Hassan M, Blanc PJ, Pareilleux A, Goma G. Production of single-cell oil from prickly-pear juice fermentation by *Cryptococcus curvatus* grown in batch culture. World J Microbiol Biotechnol. 1994; 10:534–7. doi: http://blast.ncbi.nlm.nih.gov/Blast.cgi 10.1007/BF00367661 24421128

[CR15] Kimura K, Yamaoka M, Kamisaka Y (2004). Rapid estimation of lipids in oleaginous fungi and yeasts using Nile red fluorescence. J Microbiol Methods.

[CR16] Knutsen AK, Robert V, Poot GA, Epping W, Figge M, Holst-Jensen A, Skaar I, Smith MT (2007). Polyphasic re-examination of *Yarrowia lipolytica* strains and the description of three novel *Candida* species: *Candida osloensis* sp nov, *Candida alimentaria* sp nov and *Candida hollandica* sp nov. Int J Syst Evol Microbiol.

[CR17] Kosa M, Ragauskas AJ (2010). Lipids from heterotrophic microbes: advances in metabolism research. Trends Biotechnol.

[CR18] Kumar S, Stecher G, Tamura K (2016). MEGA7: molecular evolutionary genetics analysis version 7.0 for bigger datasets. Mol Biol Evol.

[CR20] Kurtzman CP, Boekhout T, Robert V, Fell JW, Deak 
T, Boekhout T, Robert V (2003). Methods to identify yeasts. Yeasts in food: beneficial and detrimental aspects.

[CR21] Kurtzman CP, Fell JW, Boekhout T, Robert V, Kurtzman CP, Fell JW, Boekhout T (2011). Methods for isolation, phenotypic characterization and maintenance of yeasts. The yeasts: a taxonomic study.

[CR22] Kutty SN, Philip R (2008). Marine yeasts: a review. Yeast.

[CR23] Leaw SN, Chang HC, Sun HF, Barton R, Bouchara JP, Chang TC (2006). Identification of medically important yeast species by sequence analysis of the internal transcribed spacer regions. J Clin Microbiol.

[CR24] Li Q, Du W, Liu D (2008). Perspectives of microbial oils for biodiesel production. Appl Microbiol Biotechnol.

[CR25] Li M, Liu GM, Chi Z, Chi ZM (2010). Single cell oil production from hydrolysate of cassava starch by marine-derived yeast *Rhodotorula mucilaginosa* TJY15a. Biomass Bioenerg.

[CR26] Liu XZ, Wang QM, Göker M, Groenewald M, Kachalkin AV, Lumbsch HT, Millanes AM, Wedin M, Yurkov AM, Boekhout T, Bai FY (2015). Towards an integrated phylogenetic classification of the *Tremellomycetes*. Stud Mycol.

[CR27] Mamatha SS. Polyunsaturated fatty acids of *Mucor* sp. with special reference to gamma linolenic acid. Dissertation, University of Mysore; 2009.

[CR28] Martini A (1992). Biodiversity and conservation of yeasts. Biodivers Conserv.

[CR29] Mohamed MAE, Abdel-Razik AB, Ibrahim SA. Identification of different species of *Rhodotorula* using internal transcribed spacers. Open Repos Agri. 2014: e45011822. doi: 10.7392/openaccess. 45011822.

[CR30] Nigam PS, Singh A, Bat CA, Tortorello ML (2014). Fermentation (industrial) production of oils and fatty acids. Encyclopedia of food microbiology.

[CR31] Oda Y, Yabuki M, Tonomura K, Fukunaga M (1997). A phylogenetic analysis of *Saccharomyces* species by the sequence of 18S–28S rRNA spacer regions. Yeast.

[CR32] Pan LX, Yang DF, Shao L, Li W, Chen GG, Liang ZQ (2009). Isolation of oleaginous yeasts from the soil and studies of their lipid producing capacities Food Technol. Biotechnol.

[CR33] Patel A, Pravez M, Deeba F, Pruthi V, Singh RP, Pruthi PA (2014). Boosting accumulation of neutral lipids in *Rhodosporidium kratochvilovae* HIMPA1 grown on hemp (*Cannabis sativa* Linn) seed aqueous extract as feedstock for biodiesel production. Bioresour Technol.

[CR34] Ratledge C (2004). Fatty acid biosynthesis in microorganisms being used for single cell oil production. Biochemie.

[CR35] Ratledge C, Cohen Z, Ratledge C (2005). Single cell oils for the 21st century. Single cell oils.

[CR36] Sláviková E, Vadkertiová R (2003). The diversity of yeasts in the agricultural soil. J Basic Microbiol.

[CR37] Sugita T, Nishikawa A, Ikeda R, Shinoda T (1999). Identification of medically relevant *Trichosporon* species based on sequences of internal transcribed spacer regions and construction of a database for *Trichosporon* identification. J Clin Microbiol.

[CR38] Thakur MS, Prapulla SG, Karanth NG (1988). Microscopic observation of Sudan Black B staining to monitor lipid production by microbes. J Chem Technol Biotechnol.

[CR39] ThiruM Sankh S, Rangaswamy V (2011). Process for biodiesel production from *Cryptococcus curvatus*. Bioresour Technol.

[CR40] Vilgalys R, Hester M (1990). Rapid genetic identification and mapping of enzymatically amplified ribosomal DNA from several *Cryptococcus* species. J Bacteriol.

[CR41] Wang QM, Yurkov AM, Göker M, Lumbsch HT, Leavitt SD, Groenewald M, Theelen B, Liu XZ, Boekhout T, Bai FY (2015). Phylogenetic classification of yeasts and related taxa within *Pucciniomycotina*. Stud Mycol.

[CR42] White TJ, BrunsT Lee S, Taylor J, Innis N, Gelfand D, Sninsky J, White T (1990). Amplification and direct sequencing of fungal ribosomal RNA genes for phylogenetics. PCR protocols: a guide to methods and applications.

[CR43] Wynn JP, Ratledge C. Microbial production of oils and fats. In: Shetty K, Paliyath G, Pometto A, Levin RE, editors. Food biotechnology. 2nd edn. Taylor and Francis Group: New York; 2006. pp. 443-47. www.mycobank.org.

[CR44] Yarrow D, Kurtzman CP, Fell JW (1998). Methods for the isolation, maintenance and identification of yeasts. The yeasts: a taxonomic study.

[CR45] Xing D, Wang H, Pan A, Wang J, Xue D (2012). Assimilation of corn fiber hydrolysates and lipid accumulation by *Mortierella isabellina*. Biomass Bioenerg.

[CR46] Zhao CH, Chi Z, Zhang F, Guo FJ, Li M, Song WB, Chi ZM (2011). Direct conversion of inulin and extract of tubers of Jerusalem artichoke into single cell oil by co-cultures of *Rhodotorula mucilaginosa* TJY15a and immobilized inulinase-producing yeast cells. Bioresour Technol.

